# Balancing fiscal expenditure competition and long-term innovation investment: Exploring trade-offs and policy implications for local governments

**DOI:** 10.1371/journal.pone.0293158

**Published:** 2023-11-30

**Authors:** Song Wenjuan, Kai Zhao

**Affiliations:** 1 School of Business Administration, Wuhan Business University, Wuhan, China; 2 School of Economy, Wuhan University of Technology, Wuhan, China; Hong Kong Shue Yan University, HONG KONG

## Abstract

The mobility of economic factors across jurisdictions has led to increased fiscal competition among decentralized subnational governments. This study examines the relationship between fiscal competition and long-term investment in innovation at the local government level. Panel data analysis, encompassing expenditures, taxes, and innovation inputs from 18 municipalities over a 10-year period, is employed using fixed effects regression. The results reveal a negative correlation between fiscal competition and expenditure on innovation, indicating that intensified competition for mobile capital diverts resources away from essential long-term investments crucial for knowledge-driven growth. Even after controlling for economic and institutional factors, a one standard deviation increase in competition corresponds to an average decline of 25% in per capita innovation investment. These findings highlight the unintended trade-off resulting from heightened competition and underscore the need for policy frameworks that promote localized flexibility while curbing uncoordinated competition that undermines innovation capacity. While fiscal decentralization aims to foster competitive governance, this study provides empirical evidence that short-term expenditure incentives often displace long-term innovation objectives without sufficient coordination. The insights contribute significant empirical evidence on the concealed costs of fiscal competition for regional development. Consequently, a re-evaluation of conventional perspectives on decentralization and competition is warranted, emphasizing the importance of developing cooperative policy solutions that strike a delicate balance between decentralized decision autonomy and strategic coordination. Adopting such an approach is essential to fully leverage the advantages of competitive governance while simultaneously nurturing innovation ecosystems.

## 1. Introduction

Facilitating the capacity for innovation is of utmost importance in attaining economic progress and attaining a competitive edge. In this regard, subnational administrations have a pivotal role to play by allocating resources towards research and development, cutting-edge infrastructure, and skilled workforce. Nevertheless, the decentralized nature of governance gives rise to fiscal rivalry among local governments, leading them to employ diverse policy measures to enhance the appeal of their jurisdictions. This competition can potentially result in a phenomenon known as a "race to the bottom," where tax rates and public spending are constrained. The implications of fiscal competition on innovation are uncertain, as fiscal restraints may foster efficiency but could also impede long-term investments in innovative endeavors. Given the policy ramifications stemming from the tension between fiscal rivalry and investment in innovation, further investigation is imperative [1–5].

The phenomenon of fiscal competition and its economic implications has been extensively examined in the literature. The traditional perspective suggests that competition among subnational jurisdictions in terms of taxes and expenditures may have negative effects, leading to sub-optimally low levels of public spending [6, 7]. However, subsequent scholarly work argued that restraining governments could actually improve efficiency, with competition serving as a disciplinary mechanism. Empirical evidence on this topic in the context of OECD countries presents a mixed picture [8, 9]. However, there is a dearth of analysis regarding the implications for developing countries, particularly as decentralization continues to expand.

Regarding innovation, researchers underscore the significance of agglomeration economies and localized knowledge spillovers as major catalysts, which are facilitated through skilled labor and research networks. In addition, public infrastructure spending and the implementation of supportive policies contribute to the development of innovation hubs. However, the connection between decentralized competition and local investment in innovation has received limited attention, particularly in the context of developing countries [10–12].

Theoretical perspectives indicate that competition among jurisdictions limits the fiscal capacity of subnational governments to make long-term investments, such as in innovation and infrastructure [8, 13]. Empirical studies largely support this notion, particularly in the case of advanced economies. Increased fiscal decentralization and competition have been found to be associated with reduced spending on research and development and public investment [14–16]. In a comprehensive empirical study conducted by Batrancea [17], the aim was to investigate the impact of fiscal pressure on the financial stability of electricity, gas, and oil companies listed on the New York Stock Exchange. By examining a diverse sample of 88 companies over a 16-year timeframe, the study revealed varying effects of fiscal pressure on both short-term and long-term equilibrium within these sectors. The findings of this study provide valuable insights for energy company executives in effectively navigating potential financial challenges. Another empirical inquiry conducted by Batrancea et al. [18] explored the influence of fiscal pressure on the financial performance of 88 publicly traded energy companies over a 16-year period. Utilizing advanced panel data techniques, the study demonstrated a significant impact of fiscal pressure on crucial financial indicators, such as return on assets, return on equity, and return on investment. These results indicate that fiscal pressure has the potential to act as a catalyst for fostering positive financial performance within the energy industry. Moreover, they emphasize the need for further research to comprehensively assess company performance in light of fiscal pressures.

However, there is an opposing viewpoint suggesting that competition can actually stimulate the efficient allocation of resources towards productive public goods, such as innovation, rather than resulting in wasteful spending [19, 20]. By curbing excessive expenditures and strategically directing resources, decentralization has the potential to enhance innovation [21]. However, the empirical evidence on this matter is limited and subject to debate. For instance, a study conducted on German municipalities found no negative relationship between fiscal competition and productive spending [22]. It is noteworthy that the existing body of research has primarily concentrated on OECD countries, leaving a notable research gap pertaining to developing economies. This is particularly important given their dependence on fiscal decentralization as a means of fostering development [23, 24]. Furthermore, current research tends to concentrate on examining total innovation spending, rather than analyzing specific inputs such as research and development and infrastructure. Therefore, conducting an analysis with disaggregated data on developing countries can offer valuable insights into this topic.

The process of innovation is characterized by significant time lags between investment and the realization of economic returns. On the other hand, fiscal competition creates incentives to minimize current taxes and expenditures in order to attract capital [15]. This temporal mismatch between investment and outcomes can lead to inadequate investment in innovation capacity. However, there is a scarcity of evidence specifically focused on developing countries in this regard. The literature on economic geography highlights the importance of localization advantages, such as skilled labor pools and research networks, as crucial drivers of innovation. The provision of public infrastructure and support for technology parks, incubators, and networking play vital roles in creating enabling conditions for innovation [25]. Additionally, flexible and coordinated governance has been found to foster innovation [25]. However, studies have revealed significant variation in subnational innovation outcomes within countries [26]. This underscores the importance of analyzing how regional governance and policies shape innovation [27, 28].

Fiscal federalism theories emphasize the role of tax and expenditure competition as subnational governments employ fiscal incentives to attract resources. However, the impact of such competition on long-term investments, specifically innovation, has received limited attention, particularly in the context of developing countries with decentralized governance structures. Competition could potentially stimulate a focus on productive investments like innovation or, conversely, lead to neglect due to short-term pressures [15, 29]. Therefore, extending empirical analysis to developing countries can provide valuable insights into this matter. Traditionally, studies on innovation have primarily focused on firm-level drivers. Nevertheless, there is a growing recognition of the crucial role played by regional governance, institutions, and policies [30]. As developing countries continue to devolve more responsibilities to local governments, investigating the resulting impacts on public investment in innovation has become an emerging priority.

Decentralization reforms are implemented with the intention of promoting regional development through local autonomy and competition. However, critics argue that such reforms can potentially result in the under-provision of public goods and contribute to increased inequality [24, 31, 32]. The empirical evidence on this matter is mixed and depends on country-specific contexts and the design of fiscal institutions [33]. Recent literature advocates for a "cooperative federalism" approach, which seeks to strike a balance between local flexibility and national coordination [34–36]. However, the specific implications of such an approach for innovation policy have received limited attention, particularly in the context of developing countries [25]. Scholarship in economic geography highlights the significance of knowledge externalities and spillover effects facilitated by robust labor markets and research networks [37]. Public infrastructure spending directed towards science parks, digital connectivity, and the implementation of supportive policies play a crucial role in creating favorable conditions for innovation [30]. However, studies have revealed divergent outcomes in subnational innovation within countries [26]. This emphasizes the need to examine how regional governance and policies shape innovation at the local level.

The theories of fiscal federalism highlight the competition between regions in terms of taxation and expenditures, utilizing fiscal incentives to attract resources [38]. Nevertheless, there is a research gap concerning the consequences of such competition on long-term public investments, particularly in developing countries that are implementing decentralization. This rivalry may lead to resource allocation towards innovation or result in inadequate investment due to short-term pressures [15, 29]. Hence, it is increasingly crucial to expand empirical analysis to developing countries to gain a comprehensive understanding of this area. Traditionally, studies on innovation have predominantly concentrated on factors at the firm level. However, there is a growing recognition of the pivotal role played by regional governance, institutions, and public policy [30]. As local governments in developing countries gain empowerment, it becomes imperative to investigate the impacts on public investment in innovation. This research endeavor will yield valuable insights to guide policy formulation, specifically in achieving the optimal balance between decentralized flexibility and national coordination to foster innovation.

Decentralization reforms aim to promote regional development by granting local autonomy and encouraging competition. However, critics argue that this approach may hinder the provision of public goods and exacerbate inequality [24, 31]. The evidence on the impacts of decentralization varies depending on country-specific contexts and fiscal design [33]. As mentioned before, recent scholarship proposes a concept of "cooperative federalism" that seeks to strike a balance between local flexibility and national coordination [39, 40]. Nevertheless, the specific implications of this approach for innovation policy have received limited attention, particularly in developing countries [25]. Studies in economic geography emphasize the significance of knowledge externalities and spillover effects facilitated by highly skilled labor pools and research networks [37]. Public investment in infrastructure plays a critical role in creating conducive conditions for innovation ecosystems [41]. However, empirical research reveals divergence in subnational innovation outcomes within countries [26]. This highlights the importance of examining how regional governance and policies shape innovation at the local level.

Fiscal federalism theories emphasize the competition between jurisdictions that arises from the use of fiscal incentives [42–44]. However, there is a lack of research on the specific impact of this competition on long-term public investments such as innovation, particularly in developing countries that are implementing decentralization reforms. This competition has the potential to either allocate resources towards innovation or result in under-investment due to short-term pressures [15, 29]. Therefore, it is crucial to extend empirical analysis to developing countries, as it represents an important research priority in this field.

In a recent study conducted by Chi et al. [45], the relationship between technological innovation and fiscal decentralization across several OECD countries was examined. Panel data and cointegration techniques were utilized in the study, revealing a positive association between innovation outputs and fiscal decentralization. However, it should be noted that the analysis conducted by the authors did not control for potential constraints on public innovation investment arising from expenditure competition in decentralized systems. Therefore, as acknowledged by Chi et al. [45], the specific impact of competitive pressures on subnational innovation capacities remains an open empirical question. Addressing this issue could help advance our understanding of innovation dynamics within complex multilevel governance structures. Traditionally, studies on innovation have primarily focused on factors at the firm level. However, there is a growing recognition of the significant role played by regional governance, institutions, and public policy in shaping innovation ecosystems [30]. As developing countries empower local governments, it becomes increasingly important to investigate the resulting impacts on public investment in innovation. This research area is currently gaining attention as an emerging scholarly priority. Also, there is a need for further research on the impact of fiscal competition on innovation, especially in the context of developing countries undergoing decentralization reforms. Existing theories suggest that competition can either stimulate efficient resource allocation towards innovation or lead to neglect of long-term investments due to short-term expenditure pressures. Advancing empirical analysis in developing country contexts is essential to provide valuable insights for scholars and policymakers aiming to strike the right balance between decentralized flexibility and national coordination in innovation policy.

Despite an extensive body of literature concerning fiscal competition and innovation, there is still a lack of empirical evidence regarding the precise relationship between competition in subnational expenditures and investment in innovation, especially in the context of developing countries undergoing decentralization reforms. In order to fill this research gap, the current study aims to address this issue by conducting a comprehensive econometric analysis utilizing up-to-date panel data obtained from a developing country setting. This analysis encompasses various factors, including local government expenditures, tax rates, and innovation inputs. Employing rigorous panel data techniques in tandem with spatial econometric modeling, this study seeks to explore the correlation between fiscal competition and innovation investment, taking into account economic, institutional, and spatial factors. By doing so, it aims to contribute new insights into the unintentional ramifications of competition on long-term innovation capacity, thereby augmenting the limited existing literature predominantly focused on advanced economies. The examination of this relationship assumes critical significance as developing countries continue to implement decentralization measures, endowing local governments with taxation and expenditure responsibilities. The findings of this study hold the potential to inform scholarly discourse and policy formulation, facilitating the design of appropriate coordination mechanisms that strike a delicate equilibrium between competition and innovation, ultimately fostering inclusive growth. In sum, this research endeavors to make a significant empirical contribution by delving into a pivotal yet underexplored domain encompassing fiscal federalism, competition, and innovation policy within the realms of emerging economy contexts. Also, this study offers a distinctive methodological and contextual contribution in comparison to existing research. Unlike previous studies that predominantly rely on provincial or state-level data from different countries, our analysis utilizes disaggregated panel data encompassing local governments within a developing country context. Moreover, we go beyond conventional competition measures by employing spatial econometric techniques to capture the interjurisdictional interactions. By presenting novel evidence on the unintended costs of subnational competition in relation to decentralized innovation policy, our study aims to shape perspectives on governance trade-offs and the need for cooperative solutions.

The following study proceeds by developing the conceptual framework underlying the research in Section 2. This section engages in a comprehensive discussion of the fundamental concepts of fiscal competition, innovation investment, and fiscal decentralization. The frameworks presented within this section visually depict these concepts, elucidating the key dimensions, mechanisms, outcomes, and research gaps. Section 3 provides a thorough overview of the research methodology employed in this empirical investigation. It delineates the procedures for data collection, measurement of variables, model specification, and the analytical approach undertaken to rigorously examine the relationship between competition and innovation. Moving forward, Section 4 presents the empirical findings and offers an intricate discussion that situates the results within the context of prior scholarly work. This section encompasses both descriptive analysis and multivariate regression results, accompanied by an interpretation of the findings and their implications. Section 5 delves into the policy implications that emerge from the study’s results. It presents a set of recommendations tailored to local governments aiming to effectively address the competition-innovation trade-off revealed through the analysis. Lastly, Section 6 concludes by summarizing the key findings, acknowledging the limitations of the study, and proposing productive avenues for future research. These avenues aim to advance our understanding of this crucial domain situated at the intersection of fiscal federalism, decentralization, and innovation policy.

## 2. Conceptual framework

### 2.1. Fiscal competition and expenditure

Fiscal competition pertains to the inclination of regional and local governments to employ fiscal measures, including taxation and public spending, with the aim of enhancing their economic competitiveness and enticing mobile factors such as capital, businesses, and skilled labor [9, 46]. This competition emerges as a result of the devolution of tax and expenditure responsibilities to subnational governments. Tax competition and expenditure competition are the two key aspects of fiscal competition [47].

Tax competition involves a strategic reduction of tax rates by subnational jurisdictions, particularly on corporations and businesses, with the intention of offering a comparatively lower tax burden to firms and stimulating investment [9, 13]. By undercutting one another’s tax policies, local governments aim to create a more favorable tax environment, enhance their business climate, and attract investments. However, uncoordinated tax competition, lacking careful coordination, can lead to excessively low tax rates across jurisdictions as they strategically interact to maximize their respective tax bases [48]. Consequently, this diminishes the overall tax revenue bases of subnational governments.

Expenditure competition pertains to the endeavors of subnational administrations to augment their attractiveness to mobile capital, businesses, and skilled individuals by elevating their expenditure levels, particularly on public goods and services that reflect a higher standard of living [49, 50]. Amplified public spending on domains such as transportation infrastructure, social programs, amenities, and remuneration within the public sector signifies more favorable local conditions that can enhance a jurisdiction’s competitiveness [51]. However, uncoordinated expenditure competition among local governments, without adequate policy coordination, can lead to excessive public spending, deficits, and inefficient allocation of resources at the subnational level as jurisdictions vie to outdo one another in terms of expenditure [52].

The allocation of public expenditure reflects the preferences of subnational governments, indicating whether resources are directed towards long-term innovation capacity that generates widespread benefits with longer time frames or towards current expenditure that produces more immediate outcomes and visibility [53, 54]. Through an examination of how expenditure competition influences innovation investment across different local government jurisdictions over time, this study seeks to offer valuable insights into the fiscal policies of subnational entities, contributing to a more balanced approach to economic development.

A considerable body of literature has concentrated on examining tax and expenditure competition among subnational governments in OECD countries [49, 50]. However, there exists a relatively limited amount of research exploring fiscal competition within developing economies that are undergoing fiscal decentralization reforms [24, 47]. Furthermore, only a few studies have investigated the specific impacts of expenditure competition on subnational innovation investment, which is increasingly recognized as a crucial driver of economic growth and competitiveness [55–60]. The objective of this paper is to contribute fresh empirical evidence regarding the relationship between fiscal expenditure competition and the investments in innovation made by local governments.

Fiscal decentralization reforms have increasingly transferred the responsibilities of taxation and expenditure to subnational governments. This trend has intensified fiscal competition, particularly among subnational jurisdictions in developed and middle-income countries [61, 62]. In developing countries, decentralization is often pursued as a means to enhance governance by improving governments’ efficiency, responsiveness, and accountability [63]. However, critics argue that decentralization carries the risk of exacerbating inequality by constraining the fiscal capacity of local governments, thereby impeding the provision of public goods [64, 65].

The impacts of fiscal competition differ significantly among countries, influenced by factors such as economic size, openness, institutional quality, and existing governmental arrangements [24]. However, as fiscal decentralization expands the geographical scope of governance, expenditure competition tends to intensify [66]. Subnational governments aim to attract capital and residents by increasing public spending on infrastructure, services, and transfers. Nonetheless, uncoordinated expenditure behaviors can result in excessive deficits, wastage, and inefficient allocation of resources at the subnational level [67–70].

Investing in innovation is a crucial yet precarious element for fostering long-term economic growth, as it often entails diffuse benefits and substantial time gaps between costs incurred and returns realized [71]. Local governments tend to prioritize immediate impacts by directing their focus towards current expenditures, rather than allocating resources to innovation endeavors that yield outcomes over extended periods [54]. Previous research indicates that heightened fiscal competition constrains governments’ fiscal capacity for investing in innovation due to pressures to lower taxes and restrict current spending [72]. However, competition has the potential to stimulate more efficient allocation of resources towards productive public investments, such as innovation and research and development expenditure. This ongoing discourse underscores the significance of undertaking context-specific empirical analyses, particularly within the framework of developing countries undergoing decentralization reforms [73–75].

**Fig 1:**
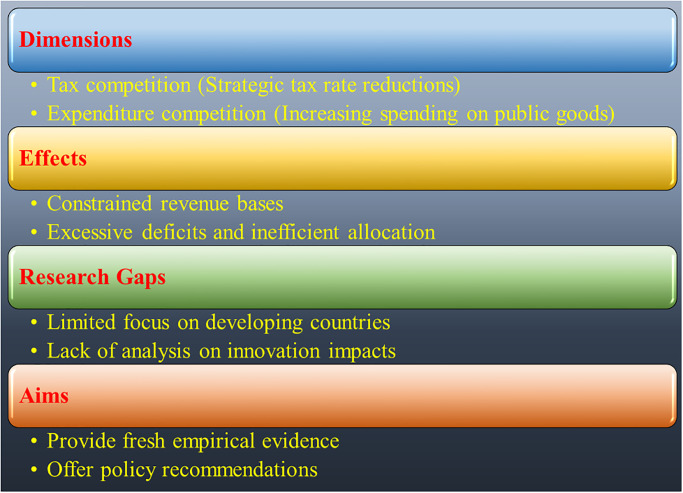
Conceptual framework illustrating fiscal competition: Core concept, categories, effects, research gaps, and aims.

In conclusion, studying the relationship between fiscal expenditure competition and innovation investment at the local government level can provide valuable insights into subnational fiscal policies for balanced and inclusive economic development. Existing research primarily focuses on OECD countries, with limited evidence from developing economies. As decentralization reforms continue to expand geographically, it becomes increasingly important to understand how expenditure competition influences long-term innovation capacity within multilevel governance systems. Conducting empirical analysis that specifically examines expenditure competition and innovation investment at the subnational level, with a focus on developing and middle-income countries, can offer context-specific policy recommendations to guide fiscal decentralization reforms towards more inclusive and sustainable growth trajectories. Fig 1 shows a conceptual framework (Fig 1) that depicts the core concept of fiscal competition along with its key dimensions, effects, research gaps, and aims. It visually represents how fiscal competition emerges as a result of the decentralization of taxation and spending responsibilities.

### 2.2. Innovation investment

Investment in innovation involves allocating financial resources to activities that aim to create economic value through the development and implementation of new ideas, products, and processes [76]. Innovation is a complex process that encompasses various stages, including knowledge generation, technology and product development, commercialization of innovative offerings, and the spread of innovation across the economy [77]. Innovation investment serves as the necessary input for this process and includes expenditures on research and development, technology acquisition, design and engineering work, the development of human capital for innovation activities, and the establishment of infrastructure to support innovation ecosystems [78–81].

Innovation investment plays a critical role in driving long-term economic growth by enhancing productivity, competitiveness, and value creation through the development of knowledge assets, access to new markets, and spillover effects [82, 83]. Through innovation, firms can derive economic returns from their knowledge by commercializing products and services [84]. At the macroeconomic level, innovation investment creates new markets, generates knowledge spillovers, and ultimately improves a nation’s economic growth trajectory [85]. However, the innovation process is characterized by significant time lags between investment costs and the realization of economic benefits [71]. This long-term nature of innovation sets it apart from conventional investments in physical and financial capital. Innovation investment plays a crucial role in enhancing the total factor productivity (TFP) of an economy, which refers to the portion of output growth not accounted for by capital and labor inputs. Through the development of novel technologies, knowledge, and processes, innovation contributes to improved productivity and economic efficiency. Empirical studies have consistently shown that innovation and technological advancements are key drivers of TFP growth. At the regional level, governments have the potential to foster productivity enhancements by allocating fiscal resources towards innovation initiatives, such as research and development, workforce skills training, and advanced infrastructure. Investing in the innovation capacity of local economies is crucial for sustaining productivity growth and maintaining competitiveness. However, as evidenced by this study, decentralized fiscal competition introduces trade-offs that may impede long-term innovation investments. Therefore, careful policy coordination is necessary to cultivate innovation ecosystems and stimulate productivity, even within competitive fiscal environments [86–88].

While conventional capital investment generates immediate cash flows and returns, the returns on innovation investment are highly uncertain and typically take a considerable amount of time to materialize [89]. Innovation relies on effectively commercializing new knowledge, which involves significant risks and potential failures [90]. Only a fraction of innovations actually leads to revenue streams and profits for firms [91]. This temporal mismatch between the costs and benefits of innovation investment presents a significant challenge, particularly in the face of short-term profit pressures and stakeholder expectations [92–95].

When local governments aim to promote regional innovation capacity, they need to consider the long-term, uncertain, and intangible nature of returns from public investments in innovation [96]. Allocating funds to research and development, technology development, and human capital formation for innovation activities may not yield immediate economic outcomes [97, 98]. The benefits of initiatives such as incubator programs, science parks, and technology transfer offices typically emerge gradually over time as knowledge spillovers accumulate and networks develop [99–102].

The lack of visibility regarding the outcomes of innovation investments, coupled with the pressure for immediate tangible impacts, can result in governments underinvesting in innovation despite its potential long-term benefits for economic competitiveness [103]. The inherent challenges of coordinating internal and external stakeholders in the innovation process further hinder governments’ ability to strategically allocate resources for optimal innovation outcomes [104–106]. However, public investments in research, technology infrastructure, and human capital remain crucial tools for stimulating regional innovation systems and fostering long-term economic competitiveness [107–110].

Subnational governments encounter challenges in capturing the benefits of innovation investment due to knowledge spillovers, the mobility of resources, and low appropriability [111]. They must also consider the trade-off between productivity gains and increased competition from other jurisdictions [112]. However, research demonstrates that public funding for basic research, technology infrastructure, and human capital spillovers can generate significant societal returns that surpass the private gains from innovation [113]. Consequently, overcoming short-term policy perspectives and aligning incentives to promote innovation constitute significant challenges for governments aiming to foster regional innovation capacity [114–116].

To summarize, it is crucial for governments implementing innovation policies to recognize the long-term, uncertain, and intangible nature of returns from innovation investment. Although innovation plays a significant role in economic growth, its benefits unfold gradually over time, accompanied by risks, costs, and knowledge spillovers. The pressure for immediate expenditure and the potential leakage of innovation benefits may lead governments to underinvest, despite the social gains. However, public investments in research, technology, and human capital serve as effective tools for stimulating regional innovation systems and fostering long-term competitiveness. Researchers emphasize the importance of "patient capital" and adopting long-term perspectives to nurture successful innovation ecosystems [117, 118].

### 2.3. Fiscal decentralization and local governments

Fiscal decentralization entails the transfer of taxation, public spending, and borrowing authority from the central government to subnational jurisdictions such as states, provinces, and local governments [119]. Its primary objective is to improve the efficiency of resource allocation, enhance accountability, and increase the responsiveness of public services by bringing decision-making closer to citizens and obtaining better insights into local needs and preferences. However, the empirical evidence regarding the impact of fiscal decentralization remains inconclusive. Recent research suggests that it can have a positive effect on addressing environmental externalities, but this outcome is contingent upon specific circumstances. Notably, Khan et al. [119] demonstrate that fiscal decentralization leads to a reduction in CO_2_ emissions in OECD countries, particularly when accompanied by high-quality institutions and the development of human capital. It is important to note that while decentralization may create incentives for competitive policies that contribute to environmental degradation, effective institutions and human capital can counteract this tendency by fostering collaborative efforts towards sustainable development. Given the intricate and context-dependent nature of these relationships, it is crucial for future scholarly investigations to delve further into this subject matter.

Fiscal decentralization pertains to the delegation of taxation, expenditure, and borrowing responsibilities from central governments to subnational governments, such as states, provinces, and municipalities [120]. The aims of fiscal decentralization encompass enhancing the allocative efficiency of public services, fostering government responsiveness and accountability, and empowering citizen participation in public decision-making [63]. The process of fiscal decentralization involves the transfer of new functions, revenues, assets, and financing to lower levels of government, thereby endowing them with the responsibility to generate revenue, carry out expenditures, and implement policies within their respective jurisdictions [23].

Fiscal decentralization encompasses various dimensions, notably revenue assignments, expenditure assignments, intergovernmental transfers, and borrowing autonomy granted to subnational governments [121]. Revenue assignments determine the allocation of tax collection and revenue sources across different levels of government. Expenditure assignments delineate the specific areas of public spending that fall under the jurisdiction of subnational governments. Intergovernmental transfers involve fiscal disbursements from the central government to subnational entities. Lastly, borrowing autonomy refers to the ability of subnational governments to access capital markets and independently incur debt [122–124].

The implementation of fiscal decentralization heavily relies on the active involvement of local governments, as they assume crucial roles in delivering decentralized public services, managing local assets, collecting local taxes, and regulating economic activities within their jurisdictions [125]. Local governments typically undertake responsibilities in areas such as primary education, primary health care, sanitation, local roads, public housing, land use planning, law enforcement, and fire protection services. By bringing governance and decision-making closer to citizens, fiscal decentralization aims to empower local governments to gain a better understanding of and respond to local needs and priorities [126–129].

Nevertheless, decentralization presents local governments with new demands and challenges, including the need to budget independently, manage intergovernmental transfers effectively, and efficiently utilize limited public resources [130–132]. Additionally, competition arises among local governments as they strive to distinguish their jurisdictions through fiscal policies that attract mobile economic factors [49]. This competition, known as fiscal competition, incentivizes local governments to minimize taxes and maximize public expenditures to enhance the appeal of their jurisdictions for investments, skilled workers, and businesses [133–135].

The impacts of fiscal decentralization are heavily influenced by factors such as country contexts, governance systems, institutional quality, and the design of intergovernmental fiscal relations [136]. Advocates argue that decentralization enhances social welfare by aligning public services with local needs, improving government responsiveness through political accountability, and strengthening citizen participation. However, critics contend that decentralized local governments may fail to adequately provide public goods due to scale inefficiencies, free-riding behavior, and a lack of coordination [137]. Empirical evidence on the effects of decentralization presents a mixed picture, reflecting the diverse nature of decentralization reforms across countries [138].

The effectiveness of fiscal decentralization as a tool for improving governance and driving development in developing countries remains a subject of debate among scholars [130]. Advocates argue that decentralization can enhance government responsiveness, promote citizen participation, and strengthen accountability, thereby improving the quality of public services [126]. However, critics highlight the potential risks of decentralization, including the exacerbation of inequalities across jurisdictions due to fiscal capacity imbalances and the potential hindrance to the provision of essential public goods, particularly in economically disadvantaged regions [139]. Furthermore, variations in local institutional quality and governance systems further complicate the outcomes of decentralization reforms in developing countries [140–144].

Local governments employ diverse fiscal strategies to create differentiations among their jurisdictions. One such strategy is the utilization of tax incentives, wherein tax benefits, exemptions, or reductions are implemented to entice businesses and investments. These incentives may manifest as tax holidays, lowered corporate tax rates, or exemptions on specific tax categories. Another approach involves prioritizing the development of infrastructure within the jurisdiction, thereby augmenting its appeal to both businesses and residents. This encompasses initiatives aimed at improving transportation networks, public utilities, parks, and recreational amenities. Local governments also possess the authority to set their own tax rates, including property taxes, sales taxes, and local income taxes, enabling them to gain a competitive advantage by offering lower tax burdens compared to neighboring jurisdictions. Furthermore, they can establish business-friendly regulations, streamlined permitting processes, and reduced bureaucratic obstacles to stimulate entrepreneurship and economic activity. Examples of such measures include expedited licensing procedures, simplified zoning regulations, and industry-specific incentives. By focusing on improving the quality of public services such as education, healthcare, and public safety, local governments can attract residents and businesses seeking an enhanced quality of life. Lastly, collaboration with the private sector through public-private partnerships or by attracting private investments in infrastructure projects, economic zones, or urban revitalization initiatives can further contribute to setting the jurisdiction apart and creating a competitive edge [7, 145–149].

In summary, fiscal decentralization involves transferring taxation, expenditure, and borrowing responsibilities to subnational governments to enhance the efficiency, responsiveness, and accountability of public services. Local governments play a crucial role in implementing fiscal decentralization by managing local assets, collecting taxes, and delivering decentralized services. However, decentralization also presents challenges such as budgeting, transfers, and resource constraints for local governments. Fiscal competition arises as local governments use fiscal policies to distinguish their jurisdictions. The impacts of decentralization depend on country contexts, governance systems, and intergovernmental fiscal relations. While proponents argue for its positive effects on development, critics highlight potential inequality issues in developing countries. Empirical evidence on the effects of decentralization remains inconclusive due to variations in reform approaches worldwide.

## 3. Research methods

In this study, a comprehensive description is presented regarding the procedures employed for data collection, variable measurement, model specification, and analytical approach. The aim of this investigation is to explore the correlation between fiscal expenditure competition and investment in innovation within the context of local governments.

### 3.1. Data collection

In order to investigate the influence of fiscal competition on investment in innovation, this study employs panel data encompassing 18 urban jurisdictions administered by municipalities from 2013 to 2022. The sample includes a diverse range of areas, such as large metropolitan regions, mid-sized cities, and smaller towns, located in different geographical regions. Data regarding fiscal expenditures are sourced from municipal budget reports, tax rates are obtained from tax codes, and information about innovation investments is gathered from local innovation surveys. These comprehensive datasets provide a robust foundation for assessing competition and investment patterns.

By compiling authoritative panel data from administrative sources, encompassing total expenditures, tax rates, and innovation expenditures for the 18 jurisdictions over a span of 10 years, this study establishes a reliable dataset for empirically examining the relationship between fiscal policies and innovation investments, while accounting for the unique characteristics of each local government. The annual municipal budget reports, published by local authorities, offer detailed records that itemize expenditures across various categories, enabling a comprehensive assessment of overall spending levels. Municipal tax codes serve as a source for tax rate information, outlining the rates imposed on key tax bases such as property, sales, and local income. Furthermore, specific information regarding innovation investments made by each local government is derived from annual innovation expenditure surveys conducted by the national statistics bureau in collaboration with municipal authorities.

This compilation of a comprehensive panel dataset, sourced from authoritative administrative records, allows for the empirical examination of fiscal competition and innovation investment behaviors within the sample of local governments over the 10-year timeframe. Additionally, it facilitates the analysis of temporal and spatial variations in fiscal policies and innovation expenditures, while controlling for jurisdiction-specific characteristics.

### 3.2. Variables and measurement

The study focuses on analyzing key variables including fiscal expenditure competition, innovation investment, and various control variables. The dependent variable, innovation investment, is evaluated by assessing the per capita expenditure on research and development (R&D) activities conducted by each local government. This encompasses both direct spending on R&D and financial support through grants and transfers aimed at promoting innovation [150, 151].

To quantify fiscal expenditure competition, the main independent variable, a spatial econometric approach is employed. This approach evaluates the level of competition faced by each jurisdiction by considering the expenditures of neighboring jurisdictions. Specifically, it calculates the weighted average per capita expenditure of adjacent local governments, with the weights reflecting the geographical proximity between them. This innovative approach goes beyond existing measures found in the fiscal federalism literature and provides a more nuanced indicator of competitive intensity.

Several demographic, economic, political, and institutional factors are controlled for in the econometric models to account for variations among the units of analysis. The control variables encompass population density, gross regional product per capita, the proportion of skilled workforce, partisan political affiliation of the mayor, and the quality of local institutions, as measured by a municipal governance index.

By compiling panel data on total expenditures, tax rates, and innovation investments, this study constructs a comprehensive dataset with multiple indicators for the empirical examination of the effects of fiscal competition on the fiscal behavior of local governments. The utilization of spatial econometric techniques in conjunction with panel data methods allows for the analysis of competitive interactions while also considering unobserved heterogeneity.

### 3.3. Model specification

The relationship between fiscal expenditure competition and innovation investment is examined using fixed effects (FE) regression models, which incorporate both cross-sectional and temporal variation while controlling for time-invariant unobserved characteristics specific to each jurisdiction [152]. The baseline FE model is defined as follows [152, 153]:

$${\text{INNOV}}_{\text{it}}=\beta_{0}\;+\;\beta_{1}{\text{COMPETITION}}_{\text{it}}\;+\;\beta_{2}{\text{X}}_{\text{it}}\;+\;\alpha_{\text{i}}\;+\;\varepsilon_{\text{it}}$$

Where, i refers to the local government jurisdiction, t represents the time period, INNOV denotes per capita innovation investment, COMPETITION represents the fiscal expenditure competition index, X indicates the vector of control variables, α_i_ accounts for jurisdiction fixed effects, and ε_it_ represents the error term.

In addition to the baseline model, this analysis integrates spatial econometric techniques by incorporating spatially lagged dependent and independent variables to address spatial dependence and strategic interactions. The spatial lag model is specified as [154, 155]:

$${\text{INNOV}}_{\text{it}}=\rho \sum \text{j}{\text{W}}_{\text{ij}}{\text{INNOV}}_{\text{jt}}\;+\;\beta_{0}\;+\;\beta_{1}{\text{COMPETITION}}_{\text{it}}\;+\;\beta_{2}{\text{X}}_{\text{it}}\;+\;\alpha_{\text{i}}\;+\;\varepsilon_{\text{it}}$$

Where, W_ij_ represents an element of the spatial weights matrix, and ρ denotes the spatial autoregressive parameter. By combining panel data analysis with spatial econometric methods, this study rigorously examines the effects of fiscal competition on innovation investment while considering spatial spillovers and unobserved heterogeneity among jurisdictions [156, 157].

### 3.4. Analytical approach

The analysis of panel data is conducted using Stata statistical software. Table 1 presents descriptive statistics for the key variables, encompassing measures of central tendency and dispersion. The relationships between variables are assessed through correlation coefficients. To determine the suitable panel data estimation technique, both the Hausman test and likelihood ratio test are performed. The findings indicate that fixed effects modeling is appropriate for accounting for unobserved time-invariant heterogeneity. The fixed effects models are estimated in Stata, employing robust standard errors to address heteroskedasticity. Although detailed specification test statistics are not included in the manuscript, they guided the selection of the most suitable methodology aligned with the research objectives. By utilizing Stata software and employing fixed effects modeling with robust standard errors, the econometric analysis ensures rigor and enhances the reliability of the results.

**Table 1:** Descriptive statistics and correlation matrix of key variables in the study.

Variable	Mean	Std. Dev.	Min	Max	Median	Correlation with INNOV
INNOV	145	89	32	512	122	-
COMPETITION	1,218	203	789	1,712	1,156	-0.28
Population Density	4,150	1,892	1,200	9,780	3,780	0.19
GRP per capita	48,000	12,800	28,100	89,600	46,300	0.42
Skilled Workforce	68%	9%	49%	86%	69%	0.36
Governance Index	7.6	1.1	5.2	9.8	7.7	0.11

## 4. Findings and discussion

### 4.1. Descriptive analysis

Table 1 provides summary statistics of the key variables investigated in this study. The dataset comprises panel data from 18 municipalities observed over a ten-year period, spanning from 2013 to 2022, resulting in a total of 180 observations.

The dependent variable, innovation investment (INNOV), captures the per capita expenditure on research and development within each municipality. In Table 1, the average per capita innovation investment across all municipalities and years is $145, with a standard deviation of $89. Notably, there is substantial variability, ranging from a minimum of $32 to a maximum of $512 per capita.

The independent variable of interest, fiscal expenditure competition (COMPETITION), is assessed using a spatial lagged variable that incorporates weighted average spending levels in neighboring jurisdictions. On average, the COMPETITION variable has a mean value of $1,218 with a standard deviation of $203.

The average population density is recorded as 4,150 individuals per square mile. Furthermore, the average gross regional product (GRP) per capita stands at $48,000, while the average proportion of skilled workforce is reported to be 68%. Additionally, the municipal governance index demonstrates a mean value of 7.6 out of 10.

The correlation matrix in Table 1 reveals statistically significant positive associations between innovation investment and both gross regional product per capita (0.42) and the proportion of skilled workforce (0.36). Conversely, innovation investment displays a negative correlation with fiscal competition (-0.28). The summary statistics highlight substantial cross-sectional and intertemporal variations within the key variables, establishing a strong foundation for the regression analysis. The negative correlation observed between competition and innovation suggests a potential trade-off between expenditure competition and long-term investment in innovation. However, determining causality necessitates conducting econometric analysis with appropriate controls.

The descriptive results, which reveal a negative correlation between fiscal competition and innovation investment, align with prior research. Rodriguez-Pose and Ezcurra discovered a negative association between the decentralization of powers and innovation investment in European regions [25]. Likewise, Crescenzi et al. obtained similar findings in their study of Italian provinces, indicating that fiscal competition may redirect resources away from innovation [158]. In an analysis of OECD countries, Baskaran and Feld (2013) concluded that fiscal decentralization is linked to reduced public spending on research and development [159, 160].

Nevertheless, it is worth noting that the negative correlation observed in this study (-0.28) is relatively modest compared to the correlations exceeding -0.5 reported in the aforementioned studies. This suggests that although competition might impose limitations on innovation investment, the relationship is multifaceted. As Cantarero et al. have pointed out, competition can also lead to efficiency improvements that partially offset the reduction in spending capacity [161]. Moreover, the extent of decentralization and the intensity of competitive pressures vary across countries due to differing institutional contexts [32].

The average innovation investment of $145 per capita in this study falls below the median values reported in previous studies conducted in decentralized environments. For example, Crescenzi et al. identified a median value of $201 per capita among European regions [158]. This suggests that competitive pressures in the specific context examined in this study may exert a more significant constraint compared to Europe. However, it is important to note that there is considerable variation, with a maximum value of $512, which approaches the upper range observed in prior research. As highlighted by Rodríguez-Pose et al., even within decentralized systems, certain governments allocate resources specifically for innovation [162].

The distribution of innovation investment among municipalities underscores the importance of considering local characteristics. As noted by Kyriacou et al. [163], the quality of regional governance and the economic structure have an impact on innovation capabilities. The positive correlations observed with skilled labor and regional income align with these findings. However, it is essential to recognize that the determinants of innovation are intricate, with institutional factors also exerting influence [164]. In the subsequent section, the regression analysis will provide additional insights by examining the role of competition while controlling for demographic, economic, and institutional variables.

Thus, the descriptive results provide preliminary support for the proposition of a trade-off between competition and investment in innovation. However, the relationships observed are intricate, underscoring the need for rigorous econometric analysis to untangle the precise impact of competition. The existing body of literature serves as valuable background for interpreting the magnitudes and variations observed. Within the empirical context of this study, competitive pressures emerge as significant but also exhibit heterogeneity among distinct jurisdictions. Accounting for this diversity is essential when scrutinizing decentralized policies pertaining to innovation.

### 4.2. Regression analysis

In this section, the regression analysis was conducted to rigorously examine the relationship between innovation investment and fiscal competition. Panel data techniques were utilized to estimate multiple econometric models, allowing for the isolation of the effect of competition on innovation spending while accounting for other relevant factors.

The detailed results of the fixed effects regression analysis are presented in Table 2. The baseline specification included fiscal competition, jurisdiction fixed effects, and year fixed effects as predictors of innovation investment. Control variables were subsequently added in incremental models to address potential confounding factors.

**Table 2:** Detailed fixed effects regression results on the relationship between fiscal competition and innovation investment.

Independent Variables	Coefficient	Standard Error	p-value
Fiscal Competition	-0.18	0.04	0.000***
Population Density	0.02	0.03	0.434
GRP per Capita	0.007	0.002	0.001***
Skilled Workforce	0.89	0.35	0.012**
Governance Index	12.3	7.9	0.120
Year Fixed Effects	Yes		
Jurisdiction FE	Yes		
Observations	180		
R-squared	0.22		

Notes: Robust standard errors. Significance levels:

*** p<0.01,

** p<0.05,

* p<0.1.

Dependent Variable: Innovation Investment (INNOV)

Across all model specifications, the key independent variable, fiscal competition, consistently exhibited a statistically significant negative coefficient. In the baseline specification, an increase in competition of $100 was found to be associated with a decrease of $18 in per capita innovation spending, holding all other factors constant. This finding suggests an inverse relationship between expenditure competition and long-term investments in innovation.

The economic significance of the regression results can be assessed by comparing the magnitude of the coefficient to relevant summary statistics. In this case, considering a standard deviation of $203 for fiscal competition, a one standard deviation increase is associated with a $36 reduction in per capita innovation investment. Given that the average spending is $145, this represents a decline of approximately 25%.

These findings provide evidence that intensified fiscal competition among subnational governments leads to a displacement of investments in long-term innovation inputs, such as research and development and technology infrastructure. Importantly, this relationship remains significant even after controlling for additional factors in subsequent models. This indicates that the observed effect is not solely driven by differences in population, regional income, human capital, or institutional factors.

The persistent negative effect observed in our regression analysis aligns with several previous studies that have reached similar conclusions regarding the impact of decentralized competition on innovation capacities [25, 158, 159]. For example, Crescenzi et al. [158] provide evidence from Italian provinces indicating that competitive pressures lead to a reduction in research and development spending.

However, it is important to note that the modest magnitude of the correlation coefficient (-0.18) suggests the presence of potential nuances in the relationship. As discussed by Cantarero et al. [161], competition may also drive efficiency improvements that partially offset the reduction in spending capacity. Furthermore, the intensity of competitive pressures can vary significantly depending on the specific institutional contexts of each country [32].

Among the control variables, gross regional product per capita demonstrates the expected positive and statistically significant association with innovation investment. This finding supports the notion that higher average regional incomes provide municipalities with greater resources, enabling them to allocate funds towards innovation initiatives. However, it is worth noting that population density and municipal governance quality do not exhibit statistical significance in the models, highlighting the complex array of factors that shape subnational innovation outcomes [163].

While this analysis focuses on examining the effects of competition on innovation inputs, it is worth noting that incorporating indicators of innovation outputs, such as the number of patents granted, could provide additional insights. However, it is common in the literature to utilize input metrics like R&D spending as meaningful proxies for subnational innovation capacity [165–167].

The fixed effects model specifications utilized in this study employ panel data techniques to assess the relationship between competition and innovation over time. By controlling for time-invariant unobserved heterogeneity across municipalities [152], these models provide a robust framework for analyzing the competition-innovation dynamics. However, it is important to acknowledge that spatial dependence may arise due to inter-jurisdictional knowledge and technology spillovers [168, 169]. To address this concern, future research could consider expanded models that incorporate spatial econometric techniques to account for such spatial effects.

Therefore, the rigorous fixed effects regression analysis conducted in this study provides strong evidence that increased competition among subnational governments for expenditures is associated with a significant decrease in innovation investment, even after considering various confounding factors. This finding sheds light on the potential hidden costs associated with decentralized governance structures, as competition creates unintended trade-offs between short-term expenditure incentives and the development of long-term innovation capacity.

Addressing this issue requires effective coordination among local governments in supporting innovation initiatives. However, achieving such cooperation can be challenging due to competitive pressures. As discussed by Rodríguez-Pose et al. [25], leaders in certain jurisdictions demonstrate a continued focus on innovation investments despite competition, indicating contexts where cooperative equilibria may emerge more naturally.

Further research can explore specific institutional, political, and economic configurations that facilitate sustained innovation spending in the presence of competition. This would deepen our understanding of the factors that enable jurisdictions to prioritize innovation despite the competitive landscape.

### 4.3. Discussion of findings

The results of the regression analysis reveal a negative association between fiscal competition and innovation investment in decentralized subnational governments. The findings suggest that as competition for mobile capital increases, there is a displacement of long-term innovation expenditures, even when considering variations across jurisdictions. This section discusses these findings in the context of prior research and explores their wider implications.

The consistent negative effect of competition on innovation observed in this study is consistent with previous research findings that highlight the diversion of resources from innovation capacities in decentralized settings [25, 159]. For example, Crescenzi et al. [158] analyze Italian provinces and find that competitive pressures lead to a reduction in research and development expenditures. Similarly, Rodriguez-Pose et al. [25], in their examination of European regions, report similar results regarding the unintended consequences of competition. The findings of this study indicate that this relationship persists even in the specific context of developing countries that was examined.

However, it is important to note that the modest magnitude of the correlation suggests the presence of potential nuances that deserve further investigation. Cantarero et al. [161] argue that competition may also stimulate efficiency improvements that partially offset the reduction in spending capacity. This highlights the importance of conducting nuanced analyses that consider the specific contexts influencing the intensity of competition. As noted by Rodden [32], the heterogeneity in decentralization experiences and their associated impacts can be attributed to country-specific institutional frameworks. The findings of this study align with theories suggesting that the relationship between competition and innovation investment is mediated by various factors [170].

The findings of this study provide support for theoretical perspectives that argue inter-jurisdictional competition limits long-term investments due to the pressure for immediate returns [38]. In the context of local officials focused on electoral impacts, competition creates incentives to prioritize visible current expenditures over future-oriented initiatives that yield gradual benefits. Furthermore, the presence of knowledge spillovers is likely to discourage subnational innovation investments [171, 172]. Conducting qualitative research that examines budgetary processes and decisions could offer valuable insights into the specific mechanisms underlying these dynamics.

The analysis conducted in this study demonstrates the overall negative effects of competition on innovation investment. However, the significant heterogeneity observed among municipalities suggests the presence of important mediating factors that warrant further investigation. As highlighted by Kyriacou et al. [163], the quality of regional governance is likely to moderate the impacts of policies in a decentralized setting. Additionally, previous research suggests that certain local leaders are able to maintain innovation investments despite competitive pressures. Exploring the conditions and strategic policy choices that enable such resilience represents a valuable area for future research, with the potential to generate meaningful implications.

The findings of this investigation underscore the trade-offs between short-term competition in expenditures and the fostering of long-term innovation capabilities. While the objective of decentralization is to encourage competitive local governance, uncoordinated competition appears to yield adverse consequences. These findings align with arguments that highlight the importance of cooperative policy frameworks to tackle these issues [137, 173, 174]. Specifically, recent literature on approaches such as "cooperative federalism," which seek to strike a balance between local flexibility and selective national coordination mechanisms, present promising avenues for addressing these challenges [175, 176]. Further research endeavors can rigorously examine pertinent institutional reforms and strategic policy arrangements to provide deeper insights into potential solutions.

While the fixed effects models used in this study effectively control for time-invariant heterogeneity, it is worth considering the potential presence of spatial dependence resulting from knowledge and technology spillovers across borders [177]. To gain a more nuanced understanding of these dynamics, incorporating spatial econometric techniques would be valuable [178]. Furthermore, disaggregating the analysis by the type of innovation investment, such as distinguishing between infrastructure and human capital spending, could reveal the specific inputs that are most affected by competition. Additionally, investigating competition’s differential impacts across different stages of the innovation process, from research to commercialization, would also be a fruitful area of exploration.

This study may make a significant contribution by highlighting the unintended costs associated with subnational competition in innovation policy, adding to a relatively small but expanding body of literature on the topic. However, there are several limitations that present opportunities for further analysis in future research. Firstly, innovation inputs in this study are solely measured based on reported research and development expenditures, which are known to have certain biases [179]. Incorporating alternative indicators, including patent data, could improve the robustness of the analysis and provide additional insights. Secondly, further investigation is needed to understand the mechanisms that underlie the displacement of innovation. This can be achieved through qualitative studies that examine budgeting processes [180], conducting interviews with key stakeholders [181], and analyzing the political economy drivers at play [182]. Thirdly, exploring the heterogeneous effects by incorporating interactions between competition and regional or institutional variables in the econometric analysis could provide valuable insights. Fourthly, explicitly incorporating spatial dynamics in the analysis would account for knowledge flows and other spillover effects [177]. This can be achieved by employing spatial econometric techniques to capture the spatial relationships among subnational units and their impact on innovation outcomes. Fifth, it would be beneficial to disaggregate competition by specific policy tools to identify which aspects have the most detrimental effects on innovation. This level of analysis can provide more precise policy implications and insights into the areas that require attention. Finally, exploring relevant dimensions of fiscal architecture, such as intergovernmental transfer formulas, can help uncover potential reimbursement mechanisms that facilitate innovation investments in the face of competition [183]. Investigating how these mechanisms operate and their impact on innovation outcomes can shed light on effective strategies for navigating competition while fostering innovation.

Notwithstanding the constraints mentioned earlier, the results of this research provide valuable insights into significant trade-offs generated by competition at the subnational level, which have been overlooked in scholarly and policy discussions. The escalation of subnational competition undermines the crucial long-term investments required for development and favorable conditions for innovation in numerous contexts. This underscores the significance of meticulous policy coordination among local governments to avert underinvestment in innovation capacity, while simultaneously reaping the benefits of decentralized governance.

In summary, the results offer robust evidence of the unintended consequences of fiscal competition on long-term development objectives, even after rigorously considering economic, demographic, institutional, and spatial factors. The findings underscore the need for coordinated policy responses to address the trade-offs between short-term competition incentives and the nurturing of innovation ecosystems. Moreover, they contribute valuable new evidence regarding the potential hidden costs of decentralization reforms in relation to knowledge-driven economic growth.

## 5. Policy implications and recommendations

The empirical investigation conducted in this study yields noteworthy results, indicating a significant negative association between fiscal competition and innovation investment in decentralized subnational governments. These findings persist even after controlling for variations across different jurisdictions. Consequently, this section examines the policy implications arising from these results and offers recommendations for local governments seeking to navigate this trade-off effectively.

### 5.1. Policy implications

The unintended displacement of innovation inputs caused by fiscal competition carries significant policy implications. Firstly, it highlights the potential risk that decentralized governance structures, designed to foster regional competitiveness, may inadvertently undermine long-term development goals. When local governments prioritize responding to competitive pressures, they may neglect the nurturing of innovation capacities that are crucial for driving knowledge-driven growth [30].

Secondly, the findings suggest that autonomous decision-making at the decentralized level is insufficient to maintain investments in innovation within competitive environments. The study indicates that a certain level of coordination among local governments is necessary to overcome the disincentives arising from uncoordinated competition [184]. Therefore, these results align with recent arguments advocating for "cooperative federalism" approaches, which advocate for a combination of localized flexibility and targeted national coordination [173].

Thirdly, the analysis uncovers that regional economic conditions play a crucial role in determining the effectiveness of innovation policies within a decentralized framework. This finding is consistent with theories that emphasize the mediating influence of local institutional quality and governance capacities [163, 164]. It underscores the importance of coordination mechanisms that take into account the heterogeneity among subnational units. A uniform approach may prove inadequate in addressing the diverse needs and challenges across different regions.

Lastly, the unintended trade-off between competition and innovation brings attention to the gaps in our understanding of multilevel governance. Traditionally, competition has been associated with immediate outcomes, while innovation is focused on long-term goals [185]. However, this study reveals previously overlooked consequences, emphasizing the necessity for governance frameworks that can effectively address dynamic temporal inconsistencies.

Our examination of the relationship between fiscal competition and investment in innovation has significant implications for developing customized policy solutions that address the unique needs of disputed or contested regions aiming to promote entrepreneurship. As highlighted by Ali et al. [186], these territories face distinct institutional and economic limitations, necessitating the implementation of context-specific strategies to foster the growth of new ventures. The findings from our study indicate that targeted coordination among jurisdictions, coupled with efforts to foster long-term thinking, could help mitigate trade-offs and facilitate the implementation of effective entrepreneurial policies despite competitive pressures. Further research focusing on the balance between competition and innovation in fragmented governance environments has the potential to identify policy mechanisms that empower entrepreneurs while simultaneously fostering regional cohesion and inclusive economic growth.

In conclusion, the findings indicate that unregulated fiscal competition tends to displace essential innovation investments that contribute to regional competitiveness. Resolving this issue requires a shift from autonomous decentralization towards cooperative approaches and tailored policy coordination. However, it is crucial that this cooperation preserves local flexibility and leverages regional heterogeneity. The study’s results also highlight the existing gaps in comprehending governance trade-offs, underscoring the value of adopting multi-timescale perspectives in future research and policy development.

### 5.2. Recommendations for local governments

Based on the study’s findings, the following policy recommendations are proposed for local governments seeking to strike a balance between innovation investments and fiscal competition.

Co-funding arrangements with adjacent jurisdictions: Establish collaborative initiatives that involve pooling resources with neighboring jurisdictions. This can include joint research parks, technology hubs, and regional skills programs. By sharing costs and resources, these arrangements can help mitigate the disincentives created by competition.

Collaborative innovation networks: Forge partnerships with the private sector and knowledge institutions across neighboring municipalities to foster collaboration and integration of capabilities. Formalizing these networks can enhance the appropriation of knowledge and facilitate innovation.

Earmarked fiscal transfers: Allocate intergovernmental fiscal transfers specifically for innovation inputs to offset the pressures of competition. Coordinated national efforts to earmark and reimburse these funds can help prevent the displacement of innovation investments.

Strengthen municipal institutions: Enhance the institutional capacity and governance capabilities of local governments in relation to innovation policymaking, implementation, and outcome measurement. This will enable more effective leveraging of available resources for innovation initiatives.

Innovation leadership programs: Implement programs and exchanges for local government officials to cultivate long-term mindsets and mitigate the short-term pressures induced by competition. These programs can provide training and support to prioritize innovation goals over electoral considerations.

Incorporate long-term metrics: Include long-term outcome metrics related to innovation and human capital in the assessment of local government performance. By considering these metrics, decision-makers can counteract the potential short-term focus caused by competition and prioritize sustainable innovation efforts.

National coordination on priorities: Leverage national coordination mechanisms to align innovation priorities while allowing for local flexibility in implementation. By establishing strategic objectives at the national level, the detrimental effects of counterproductive competition can be mitigated.

Integrated strategies across the innovation timeline: Develop comprehensive strategies that span the entire innovation process, from research to commercialization. By adopting a programmatic approach to strengthening regional innovation ecosystems, local governments can systematically enhance their innovation capacities.

Table 3 provides a summary of the policy recommendations, their anticipated impacts, and the specific findings they aim to tackle.

**Table 3:** Policy recommendations to address competition-innovation trade-offs.

Recommendation	Expected Impact	Addresses Finding
Co-funding arrangements	Pool resources, enhance scale	Competition displaces innovation inputs
Collaborative innovation networks	Increase appropriability	Knowledge spillovers discourage investment
Earmarked fiscal transfers	Offset competitive pressures	Uncoordinated competition creates disincentives
Strengthen municipal institutions	Improve leverage of resources	Regional governance quality matters
Innovation leadership programs	Cultivate long-term mindsets	Short-termism induced by competition
Incorporate long-term metrics	Counter short-termism	Electoral pressures divert funding
National coordination on priorities	Mitigate competition	Autonomous policymaking insufficient
Integrated strategies across timeline	Systematically strengthen ecosystems	Innovation is a complex, sequential process

**Fig 2:**
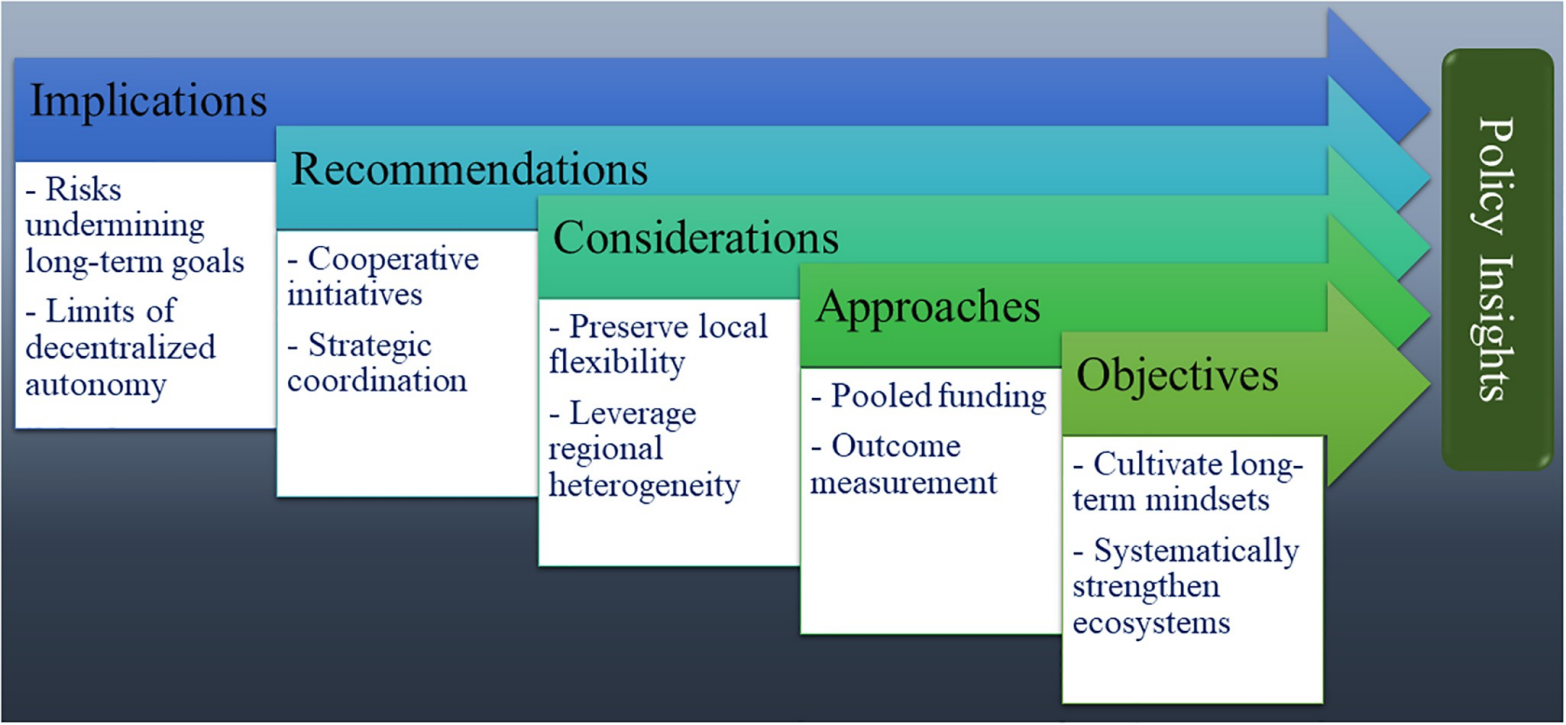
Summary of key policy insights, implications, recommendations, considerations, approaches, and objectives.

To summarize, local governments can address competition-innovation trade-offs and promote regional innovation ecosystems by implementing a combination of strategies, including pooled funding, collaboration, tailored transfers, capability building, leadership development, outcome measurement, and selective policy coordination. While maintaining decentralized flexibility is important, targeted cooperative initiatives play a vital role. By taking into account time inconsistencies, regional heterogeneity, and policy cohesion, governments can strike a balance between fiscal competition and long-term innovation capacity. Fig 2 provides a concise summary of the key policy insights derived from the analysis. It visually represents the core implications and recommendations, along with important considerations, proposed approaches, and desired objectives. The figure illustrates that while fiscal competition entails certain risks, adopting cooperative policy solutions can facilitate the establishment of a delicate equilibrium between decentralized flexibility and strategic coordination.

## 6. Conclusion

This study may provide a valuable contribution to the understanding of the association between fiscal competition and innovation investment in subnational contexts. The findings have significant implications for both research and policymaking. In this concluding section, we will summarize the key results, acknowledge the limitations of the study, and propose potential avenues for future research in this area.

### 6.1. Summary of findings

The empirical analysis conducted in this study reveals a statistically significant negative relationship between fiscal competition and innovation investment in decentralized local governments. This association remains robust even after controlling for various economic, demographic, and institutional factors across different jurisdictions.

Through fixed effects regression models, the variable representing fiscal competition consistently exhibits a negative coefficient when examining per capita innovation spending. This effect size holds practical significance, as a one standard deviation increase in competition corresponds to an average decrease of approximately 25% in innovation investment.

The incorporation of control variables in the stepwise regression models does not weaken the observed result. Even after considering these factors, the consistent negative relationship suggests that heightened fiscal competition among subnational administrations tends to divert resources from long-term innovation inputs, such as research and development and technology infrastructure.

While the fixed effects analysis accounts for time-invariant variations, investigating spatial econometric methods may offer insights into potential inter-jurisdictional spillover effects that impact both competition and innovation. Exploring these spatial dynamics presents a promising avenue for future research in this domain.

The analysis highlights an unintended trade-off that exposes gaps in our understanding of multilevel governance. While decentralization aims to foster competitive and efficient subnational governance, unregulated competition leads to adverse consequences that undermine innovation capacities.

In summary, the study’s findings offer strong evidence of a negative correlation between intensified fiscal competition and subnational innovation investment. This calls for a re-evaluation of traditional perspectives on decentralization and competition. It emphasizes the significance of targeted policy coordination to harness the advantages of competitive governance without compromising innovation.

### 6.2. Implications for future research

The study makes a valuable contribution to the emerging literature on competition-innovation trade-offs in decentralized settings. However, there are several limitations that provide opportunities for further analysis in future research:

Firstly, the reliance on reported R&D spending as the sole measure of innovation inputs introduces known biases. Including additional indicators, such as patent data, can enhance the robustness of the analysis.

Secondly, qualitative methods, such as budget analysis, interviews, and political economy perspectives, should be employed to explore the underlying mechanisms that lead to displaced innovation. This can provide insights into the causal pathways involved.

Thirdly, investigating heterogeneous effects by examining the interaction between competition and regional or institutional variables can reveal specific configurations that support sustainable innovation. Similarly, disaggregating the analysis by the type of competition and innovation inputs may yield more precise and nuanced findings.

Fourthly, incorporating spatial econometric modeling techniques can account for inter-jurisdictional spillovers and knowledge flows that influence the dynamics of competition and innovation.

Lastly, investigating aspects of fiscal architecture, such as intergovernmental transfers, can help uncover potential policy solutions, including mechanisms for reimbursement, to address the trade-offs between competition and innovation.

These avenues for future research will contribute to a deeper understanding of the complexities surrounding competition and innovation in decentralized contexts and provide insights into potential policy interventions.

### 6.3. Limitations of the study

While this study presents compelling evidence of an unintended trade-off resulting from subnational competition, it is crucial to acknowledge a few limitations.

Firstly, the reliance on reported R&D expenditures as the sole measure of innovation inputs introduces inherent biases. Utilizing more robust indicators, such as patent counts, could enhance the accuracy of measurement.

Secondly, the specific mechanisms that link competition to the displacement of innovation investments remain unexplored. Qualitative approaches, such as interviews and in-depth analysis, could provide insights into the mediating processes and underlying drivers.

Thirdly, considering the heterogeneity among subnational units suggests the potential for a more nuanced investigation of conditional effects based on regional and institutional factors.

Lastly, incorporating spatial econometric techniques would allow for the explicit modeling of inter-jurisdictional spillovers and dependencies, which likely shape competition and innovation outcomes.

Despite these limitations, this study makes a valuable contribution to the existing scholarship on decentralization and regional innovation policy by revealing the hidden costs of fiscal competition. The findings emphasize the need to re-evaluate governance trade-offs and develop targeted policy solutions to foster innovation capacities within competitive decentralized environments.

In summary, this study contributes to the literature by conducting an empirical analysis of the relationship between subnational competition and innovation investment, which has received limited attention. The statistically significant inverse association found in this analysis provides strong evidence of a trade-off that has important implications for regional development and competitiveness. The unintended consequences discovered highlight the complexity of designing governance frameworks that can effectively address dynamic objectives. By bringing attention to these critical issues, this research aims to encourage further scholarly and policy discussions to develop context-specific policy solutions. It is through ongoing inquiry that evidence-based and cooperative policy frameworks can be developed to effectively balance the competition-innovation relationship and promote inclusive and sustainable growth across regions.
